# 

**Published:** 1874-08

**Authors:** 


Pamphlets Received.—Diseases of the Conjunctiva. By Dudley S. Reynolds, M.D. Louisville, Ky., 1874. Pp. 13.

Resume of the Modern Views of the Pathology and Treatment of Glaucoma. By C. S. Fenner, M.D. Louisville, Ky. 1874. Pp. 13.

Rare Cases of Congenital Syphilis. By L. D. Bulkley, M.D. New York. 1874. Pp. 19.

Cerebro-Spinal Meningitis. By E. S. Gaillard, M.D. Louisville, Ky. 1874. Pp. 12.

Hints in the Obstetric Procedure. By W. B. Atkinson, M.D. Philadelphia. 1874. Pp. 28.

Transactions of the Medical Society of the District of Columbia. Washington. July, 1874. Pp. 48.

Annual Report of the Baltimore Eye and Ear Institute. By Julian S. Chisolm, M.D. Baltimore. 1874. Pp. 24.

### ACKNOWLEDGMENTS.

Atlanta Daily Herald; Rome Daily Commercial; American Journal of Pharmacy; American Practitioner; American Medical Weekly; Archives of American Medicine and Surgery; Boston Medical and Surgical Journal; Boston Journal of Chemistry; Baltimore Physician and Surgeon; Cincinnati Lancet and Observer; The Clinic; Chicago Medical Journal; Charleston Medical Journal; Detroit Review of Medicine and Pharmacy; Druggists' Circular; The Druggist; The Galaxy; Herald of Health; Indiana Journal of Medicine; Journal of Materia Medica; London Lancet; Medical Examiner; Medical Record; Medical News and Library; Medical and Surgical Reporter; Medical Times; Nashville Journal of Medicine and Surgery; North-Western Medical and Surgical Journal; New Orleans Medical and Surgical Journal; Pacific Medical and Surgical Journal; Popular Science Monthly; Physician and Pharmacist; Psycological & Medico-Legal Journal; St. Louis Medical and Surgical Journal; Supplement to Medical News and Library Texas Medical Journal; Virginia Medical Monthly; Western Lancet.

